# Oxygen transfer rate during the production of alginate by *Azotobacter vinelandii *under oxygen-limited and non oxygen-limited conditions

**DOI:** 10.1186/1475-2859-10-13

**Published:** 2011-02-27

**Authors:** Esteban Lozano, Enrique Galindo, Carlos F Peña

**Affiliations:** 1 Departamento de Ingeniería Celular y Biocatálisis, Instituto de Biotecnología, Universidad Nacional Autónoma de México, Apdo. Post. 510-3 Cuernavaca, 62250, Morelos, México

## Abstract

**Background:**

The oxygen transfer rate (OTR) and dissolved oxygen tension (DOT) play an important role in determining alginate production and its composition; however, no systematic study has been reported about the independent influence of the OTR and DOT. In this paper, we report a study about alginate production and the evolution of the molecular mass of the polymer produced by a wild-type *A. vinelandii *strain ATCC 9046, in terms of the maximum oxygen transfer rate (OTR_max_) in cultures where the dissolved oxygen tension (DOT) was kept constant.

**Results:**

The results revealed that in the two dissolved oxygen conditions evaluated, strictly controlled by gas blending at 0.5 and 5% DOT, an increase in the agitation rate (from 300 to 700 rpm) caused a significant increase in the OTR_max _(from 17 to 100 mmol L^-1 ^h^-1 ^for DOT of 5% and from 6 to 70 mmol L^-1 ^h^-1 ^for DOT of 0.5%). This increase in the OTR_max _improved alginate production, as well as the specific alginate production rate (SAPR), reaching a maximal alginate concentration of 3.1 g L^-1 ^and a SAPR of 0.031 g _alg _g _biom_^-1 ^h^-1 ^in the cultures at OTR_max _of 100 mmol L^-1 ^h^-1^. In contrast, the mean molecular mass (MMM) of the alginate isolated from cultures developed under non-oxygen limited conditions increased by decreasing the OTR_max_, reaching a maximal of 550 kDa at an OTR_max _of 17 mmol L^-1 ^h^-1 ^. However, in the cultures developed under oxygen limitation (0.5% DOT), the MMM of the polymer was practically the same (around 200 kDa) at 300 and 700 rpm, and this remained constant throughout the cultivation.

**Conclusions:**

Overall, our results showed that under oxygen-limited and non oxygen-limited conditions, alginate production and its molecular mass are linked to the OTR_max_, independently of the DOT of the culture.

## Background

Alginates form an important family of biopolymers. These linear polysaccharides consist of chains of (1-4)-β-D-mannuronic acid and its epimer, α-L-guluronic acid. Depending on the arrangement of these two monomers within the polymer, the properties of alginate differ strongly, thus offering a wide range of industrial applications [1]. Alginates are used mainly in the food and pharmaceutical industries as stabilising, thickening, gel- or film-forming agents; also, there are still new applications being discovered such as that of source for soluble fiber [2].

At present, the extraction from marine brown algae is the only economic way to produce alginate. However, these alginates do not meet the requirements of some specific applications in the pharmaceutical field, where polymers of a very well defined composition concerning molecular mass, composition of G- and M-blocks and block distribution, are necessary. These specifications require an important effort in downstream processing, which cannot be achieved with algal alginates [1,3].

As an alternative to the algal production process, alginate can also be produced by *Azotobacter vinelandii*, a gram-negative non-pathogenic soil bacterium. Fermentation processes generally offer the advantage of well defined production conditions, but it is still necessary to identify the effect of culture conditions on the quality and the quantity of the produced alginate. The applicability of alginate as a thickening or gel-forming agent is strongly determined by its rheological characteristics, such as the viscosity. These characteristics in turn largely depend on the molecular mass, the sequential variation and length of M- and G-blocks and the acetylation degree of alginate [4,5].

In order to be able to selectively manipulate these characteristics and thus produce alginates with well defined properties, several research efforts have been conducted [1,3,6,7]. These studies have focused on fermentation parameters such as dissolved oxygen tension (DOT), culture media composition, agitation rate and influence of CO_2 _[3,8-15].

Previous studies [9-11] have revealed the importance the DOT and the agitation rate on alginate production and its molecular weight. For example, Peña et al [9] reported that under high DOT (5% of air saturation), the bacteria produced more alginate (4.5 g L-1) than that obtained (1.0 g L-1) at low (0.5%) oxygen tension in cultures conducted at 300 rpm. On the other hand, under constant DOT (3%), increasing the agitation rate (from 300 to 700 rpm), increased the specific growth rate and the alginate production rate. However, a low agitation speed (300 rpm), leads the culture to produce a polymer of high molecular weight (680 kDa), whereas a low molecular weight (352 kDa) alginate was isolated from cultures conducted at high (700 rpm) agitation rate. Sabra et al [10] reported that in continuous culture, the alginate production rate by *A. vinelandii *increased as a function of the DOT of the culture, obtaining a specific alginate production rate of 0.2 g _alg _g _biom_^-1 ^h^-1 ^at a dilution rate of 0.22 h^-1 ^at 5% of DOT [10]. Furthermore, in the same study, the authors reported that both the molecular mass and the L-guluronic acid content increased with the DOT, reaching a maximal molecular mass of 800 kDa and a guluronic acid content of 50% in the cultures conducted at 10% of air saturation. However, in none of studies quoted above, the oxygen transfer rate has been measured and its relationship with the synthesis of alginate has been evaluated. Therefore, this is the first time that a study addressing that objective is reported.

Regarding the oxygen transfer rate (OTR), there are some reports [6,7,16,17] documenting how alginate production and the molecular mass of the polymer can be affected by the OTR and OTR_max _of the culture. Two of these studies [6,17] were made in shake flasks, where the dissolved oxygen tension was not controlled and the hydrodynamics conditions are very different to those occurring in stirred bioreactors. It has been found, that decreasing the shaking frequency in the cultures, from 200 to 100 rpm, the OTR_max _decreased from 6 mmol L^-1 ^h^-1 ^to 2.6 mmol L^-1 ^h^-1^. At lower OTR_max_, culture broths having a higher viscosity were obtained and this was associated to a higher molecular mass and acetylation degree of the polymer [17].

The other reports [7,16] have carried out studies in batch and chemostat cultures in which the oxygen transfer rate (OTR) was measured under different agitation rates and the effects caused on the molecular mass of the alginate have been evaluated. These authors have found that the alginate yield and the molecular mass of the polymer were linked to the OTR of the culture. According to those authors, the mean molecular mass of the alginate increased as OTR_max _decreased. They reported that the molecular mass obtained at 3.0 mmol L^-1 ^h^-1 ^was 7.0 times higher (1560 kDa) than at 9.0 mmol L^-1 ^h^-1 ^(220 kDa). However, in both of the previously quoted studies, the DOT was not controlled and therefore this parameter was nearly zero throughout the culture, hence all the cultures were oxygen-limited. It is important to point out that the cellular metabolism shows important differences under limited and non-limited oxygen conditions. Oxygen limitation can affect the respiratory capacity of the cells and under such conditions, the carbon source is only partially oxidized, which forces the cells to follow anaerobic pathways with the consequent production of by-products, such as acetate and lactate.

In summary, no systematic study has been reported regarding the independent influence of OTR_max _and DOT on alginate production and on the molecular mass of the alginate produced by *A. vinelandii*. In this paper, we report a study about alginate production and the changes of the molecular mass of the polymer produced by a wild-type *A. vinelandii *ATCC 9046 in terms of the maximum oxygen transfer rate (OTR_max_) in cultures where the DOT was constant.

## Results

### Analysis of the oxygen limitation conditions in the cultures

Oxygen limitation in the present study was defined in terms of the critical oxygen concentration (COC) for bacterial growth. This was defined as the DOT at which the specific growth rate of *A. vinelandii *corresponds to the 95% of the μ_max_. Previously, our group had reported that there is an important influence of the DOT on the growth rate of *A. vinelandii *in the range of 0.5 to 5% of DOT [9,13]. From these data, a maximal specific growth rate of 0.25 h^-1^, a K_s _= 1.22% of DOT (0.08 mg/L), and COC of 4% DOT (0.26 mg/L) were calculated. Therefore, in the present study, the cultures developed at 0.5% of DOT were considered oxygen-limited and the cultures at 5% were non oxygen-limited.

### DOT control at 0.5% (oxygen-limited) and 5% (non oxygen-limited)

**Figure 1:**
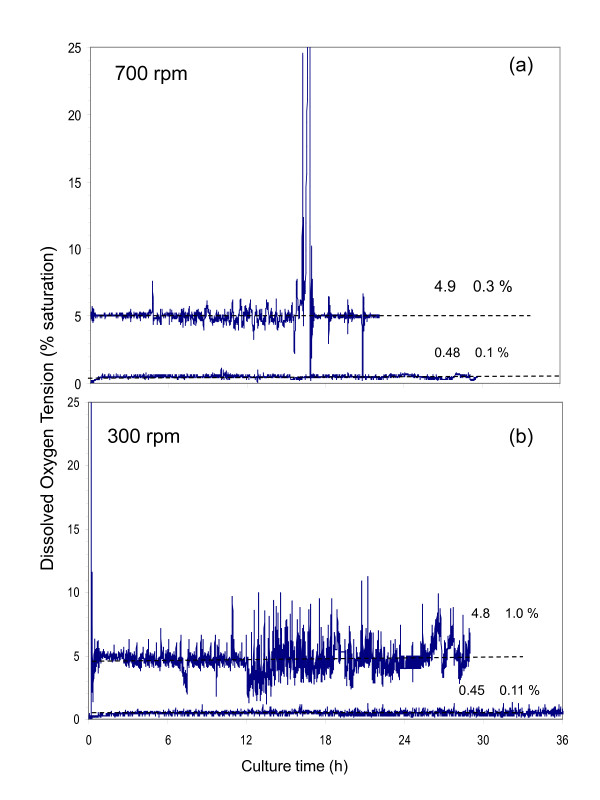
**Dissolved oxygen tension profiles of the cultures of *Azotobacter vinelandii *grown in a 3.0 L bioreactor using an airflow rate of 1.0 L min-1 at different agitation rate: (a) 700 rpm and (b) 300 rpm**.

**Figure 2:**
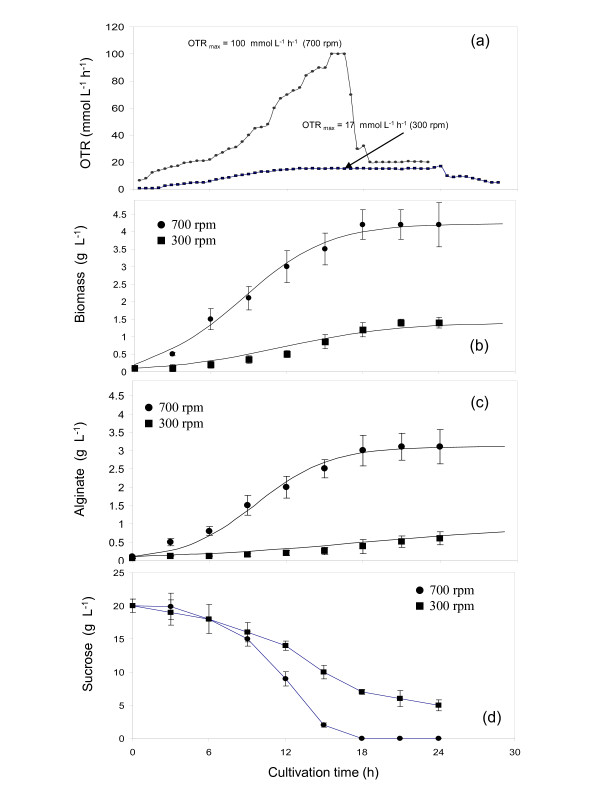
**Evolution of the oxygen transfer rate (a), cell growth (b), alginate production (c) and sucrose consumption (d) in cultures of *Azotobacter vinelandii *grown under non oxygen-limited conditions (5%) at 300 (■) and 700 rpm (●)**. Cultures were kept at a temperature of 29°C and the airflow rate was of 1.0 L min^-1^.

As shown in Figure 1, the DOT for the agitation rates evaluated (300 and 700 rpm) was controlled satisfactorily. For the cultures conducted at 700 rpm, the average DOT was 4.9 ± 0.3% between 0 and 16 h of cultivation. After that time, the DOT increased dramatically, due to the depletion of the carbon source, as shown in figure 2d. In the case of the cultures developed at 300 rpm, the average of DOT was of 4.8 ± 1.0% in the period from 0 to 26 h. Although the control at 0.5% of DOT was less accurate for both conditions tested, the DOT control was acceptable, obtaining a value of 0.48 ± 0.1% and 0.45 ± 0.11% for 700 and 300 rpm, respectively. Therefore, for all of the conditions tested, it was possible to discriminate between the influence of oxygen in the bulk liquid and the agitation rate, on the kinetics of alginate production.

### Cultures under non-oxygen limited conditions (5%)

Figure 2 shows the profiles of the oxygen transfer rate (OTR) (a) and biomass growth (b), as well as the alginate production (c) and sucrose consumption (d) of cultures of *A. vinelandii *conducted under non oxygen-limited conditions (5%), at agitation rates of 300 and 700 rpm. Although the DOT was the same under both conditions, the evolution of the OTR and the OTR_max _were completely different. These results reveal that in the cultures conducted at high agitation rate (700 rpm), the oxygen transfer rate increased exponentially during the first 16 h of cultivation, reaching a maximal value of 100 mmol L^-1 ^h^-1^. After 16 h, the OTR dropped drastically to a minimal value of 20 mmol L^-1 ^h^-1^. This behavior is typical of a limitation of the carbon source [18-20], as when the carbon source is exhausted, *A. vinelandii *decreases its respiration and therefore, the oxygen transfer rate. This is supported by the depletion of the sucrose (Figure 2d) and with the important temporal shift in the DOT (Figure 1a). As a consequence, cellular growth and alginate production stopped (Figure 2b and 2c).

In contrast, in the cultures developed at 300 rpm and 5% of DOT, the OTR increased linearly during the first hours of cultivation, achieving a maximum of 17 mmol L^-1 ^h^-1 ^at 12 h (Figure 2a). Between 12 and 24 h of cultivation a plateau was observed. This plateau of the oxygen transfer rate represents the maximum oxygen transfer capacity of the system and is a characteristic signal of oxygen limitation in the cultures [18,19].

After 24 h, a decrease from 17 to 9 mmol L^-1 ^h^-1 ^was observed, which suggest a substrate limitation [18-21]. Sucrose was not the limiting substrate, as a concentration of 5 g L^-1 ^was measured at 24 h of cultivation (Figure 2d). It might be possible that nutrients such as phosphate or trace elements could have exhausted at that point and led to a substrate limitation.

The influence of the agitation rate on the biomass growth rate, alginate production and sugar consumption is shown in Figure 2 b-d. Although the DOT was kept constant at 5% in the bulk liquid, the specific growth rate μ, (calculated using the logistic model) and the alginate production, increased when increasing the agitation rate. In the cultures conducted at 700 rpm, the specific growth rate was 0.25 h^-1 ^and the biomass concentration at the end of the cultivation was 4.2 g L^-1^; whereas at 300 rpm, μ was 0.15 h^-1 ^with a maximal biomass concentration of 1.4 g L^-1 ^(Figure 2b). A high agitation rate (700 rpm) promoted alginate synthesis, reaching a maximal concentration of 3.1 g L^-1 ^at 24 h of cultivation; whereas in the cultures developed at 300 rpm, the maximal alginate concentration was of 0.6 g L^-1 ^(Figure 2c).

As shown in Figure 2d, under high agitation rate, and therefore high OTR_max _(reached at 16 h of cultivation), sucrose was completely used by *A. vinelandii*, and the specific sucrose uptake rate was of 0.24 g _suc _g _biom_^-1 ^h^-1^. As it was mentioned above, this behaviour is linked to the drastic increase in the DOT signal and to a sharp decrease in the OTR of the culture. For the case of the cultures developed at 300 rpm, only 75% of sucrose was used by the bacteria, calculating a SSUR of 0.44 g _suc _g _biom_^-1 ^h^-1^.

### Cultures under oxygen-limited conditions (0.5% of DOT)

**Figure 3:**
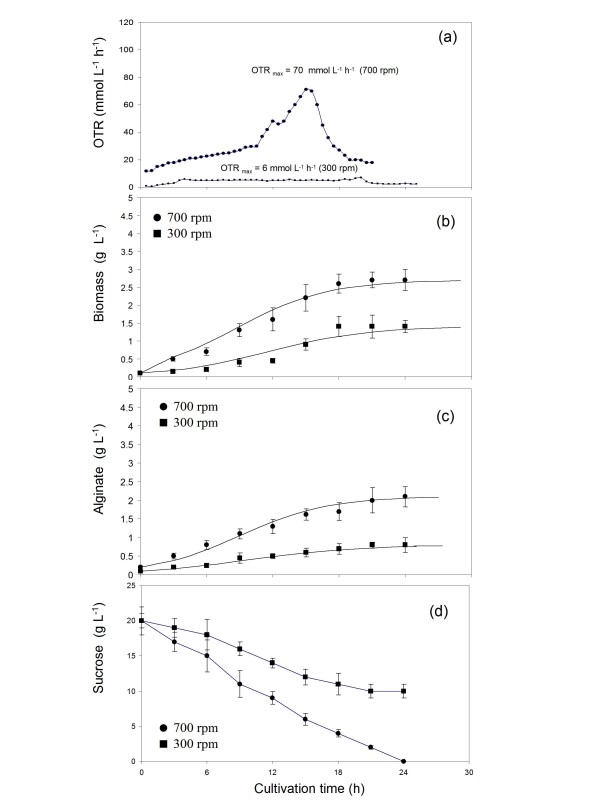
**Evolution of the oxygen transfer rate (a), cell growth (b), alginate production (c) and sucrose consumption (d) in cultures of *Azotobacter vinelandii *grown under oxygen-limited conditions (0.5%) at 300 (■) and 700 rpm (●)**.

Alginate production kinetics was also studied under oxygen limited conditions (0.5%) maintaining constant the agitation rate at 300 and 700 rpm. Similar to the results obtained in the cultures conducted at 5% (without oxygen limitation), the OTR_max_, biomass growth rate and alginate production increased when increasing the agitation rate (Figure 3 b and 3c). It is interesting to note that the OTR profile for the cultures at 700 rpm had two stages: in the first stage (0-9 h of cultivation) a linear increase in the OTR from 10 to 30 mmol L^-1 ^h^-1 ^was observed. Later, in a second stage, the OTR increased exponentially to reach a maximum of 70 mmol L^-1 ^h^-1 ^at 15 h of cultivation (Figure 3a). This behaviour seems to be typical of cultures non-limited by oxygen, such as it has been shown for other microbial cultures [20,21]. After 15 h of cultivation the OTR decreased to values close to 20 mmol L^-1 ^h^-1^. In contrast to what was observed in the cultures at 5%, this drop in the OTR is not due to the depletion of the carbon source, as at this time the sucrose concentration was approximately 7 g L^-1 ^(Figure 3 d). Analyzing the concentration of ammonium acetate used as nitrogen source, a total depletion of ammonium was observed at 15 h, which supports the fact that the culture could be limited by the nitrogen source after 15 h of fermentation. When *A. vinelandii *was cultured at 300 rpm, the OTR profile shows a characteristic plateau of the cultures with oxygen limitation, with a maximal OTR of 6 mmol L^-1 ^h^-1 ^(Figure 3a). This value of OTR remained constant from the 6^th ^to the 21^st ^h of cultivation.

As shown in Figure 3b and 3c, under the highest agitation rate (700 rpm) and therefore a higher OTR_max_, the growth rate and alginate production were higher with respect to those obtained from cultures at 300 rpm and low OTR. A maximal biomass concentration of 2.7 g L ^-1 ^and alginate production of 2.1 g L ^-1 ^were reached at 700 rpm after 21 h of cultivation. On the other hand, in the cultures conducted at 300 rpm, the biomass concentration was of 1.3 g L^-1 ^and the alginate concentration was of 0.8 g L ^-1^. In the cultures at 700 rpm the sucrose was completely consumed after 24 h of cultivation; whereas in the case of the cultures at 300 rpm 50% of initial sucrose was not consumed by that time.

### Evolution of mean molecular mass of the alginate

**Figure 4:**
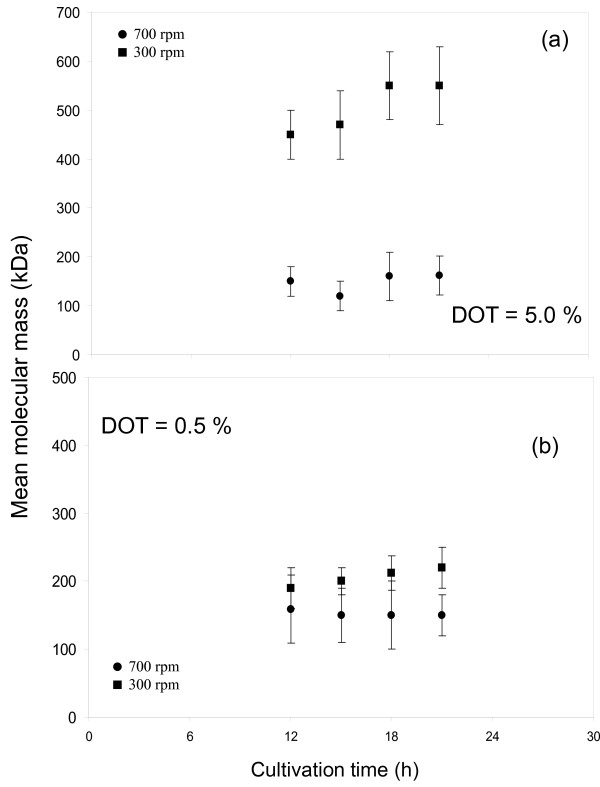
**Evolution of the mean molecular mass of the alginate produced under oxygen-limited (0.5% ) and non oxygen-limited (5%) conditions at 300 (■) and 700 rpm (●)**.

The evolution of the mean molecular mass of the alginate (MMM) produced by *A. vinelandii *during the course of the fermentation at 300 and 700 rpm is shown in Figure 4. The evolution and the maximal molecular mass (MMM_max_) was completely different for the cultures developed under the two different conditions tested. The MMM of the alginate isolated from cultures at 300 rpm and 5% (non oxygen-limited conditions) increased slightly during the culture, reaching a maximum of 550 ± 120 kDa at 21 h of cultivation; whereas the maximum MMM of the polymer produced at 700 rpm was 160 ± 40 kDa. On the other hand, under oxygen limitation (0.5%) the molecular mass of the alginate was practically the same at 300 and 700 rpm, having a MMM around of 175 ± 40 kDa and this remained constant throughout the cultivation (Figure 4 b).

**Figure 5:**
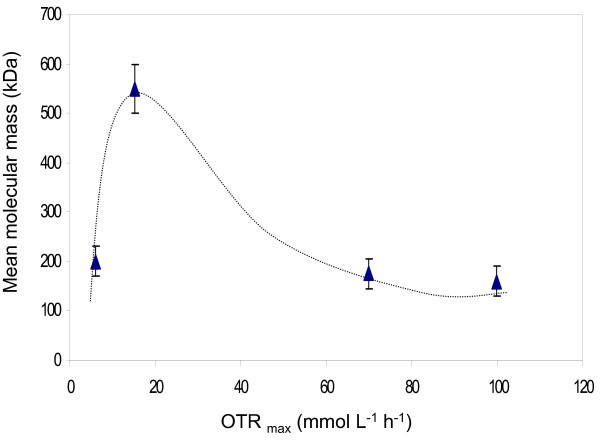
**Mean molecular mass of the alginate in terms of the maximum oxygen transfer rate (OTR_max_) for cultures developed under oxygen-limited (0.5%) and non oxygen-limited (5%) conditions**.

Figure 5 shows the mean molecular mass (MMM) of the alginate obtained at the end of the cultivation (21-24 h) in terms of the maximum oxygen transfer rate, keeping constant the DOT at 0.5 and 5%. The highest MMM (550 kDa) was reached at an OTR_max _of 17 mmol L^-1 ^h ^-1^. At low (6 mmol L^-1 ^h ^-1 ^) and high (70-100 mmol L^-1 ^h ^-1 ^) OTR_max _the MMM of the alginate was low, having values between 150 to 200 kDa.

## Discussion

Results of this study have shown for the first time that the synthesis and molecular mass of the alginate produced by *Azotobacter vinelandii *are closely associated to the maximum oxygen transfer rate (OTR_max_), in cultures where the DOT was kept constant independently of the agitation rate. The highest alginate concentration (3.1 g L^-1^) and the maximum specific alginate production rate (0.031 g _alg _g _biom_^-1 ^h^-1^) were reached in the cultures at an OTR_max _of 100 mmol L^-1 ^h^-1 ^(Figure 2). As the DOT was kept constant in the two conditions tested (300 and 700 rpm), the changes in the kinetic parameters are closely related to differences in the OTR profiles generated under the two conditions evaluated. The constant OTR profile obtained from the cultures at 300 rpm (Figure 2a), is characteristic of an oxygen limitation between 12 and 24 h of cultivation. In contrast, the behaviour observed in OTR in the cultures at 700 rpm is characteristic of the cultures where oxygen is not limiting [19].

As it was mentioned in the introduction, there are previous reports about the influence of OTR on the alginate production and its molecular mass, both in shake flasks and in stirred bioreactor cultures [6,7,16,17]. However, this is the first time that a study is reported regarding alginate production and molecular mass in terms of the OTR_max _in cultures where the DOT was maintained constant along the culture. This strategy allows evaluating only the effect of OTR_max_, independently of the other fermentation variables.

A previous study carried out in shake flasks [6], showed that both cell growth and alginate concentration (after 72 h of cultivation) were very similar in cultures developed under conditions leading to different OTR_max_. In contrast, Díaz-Barrera et al [7], using batch cultures without DOT control, found that higher OTR_max _(from 3 to 9 mmol L^-1 ^h^-1 ^) resulted in an increase the alginate concentration and alginate yield (Y _alginate_/_sucrose_) from 0.5 to 1.5 g L^-1 ^and 0.05 to 0.1 _g __alginate_/_g __sucrose _, respectively.

It is important to point out that our results contrast with those reported for the synthesis of other biopolymers, such as the case of xanthan [22]. Amanullah et al [22] studied the influence of the agitation rate (500 and 1000 rpm) and therefore the oxygen transfer rate of the culture, maintaining constant the DOT at 20% of air saturation using gas blending. According to such authors, the biological performance of the culture was independent of the agitation rate as long as the broth homogeneity could be ensured. Both the biomass concentration profile and the xanthan production were very similar in the cultures conducted under the two agitation conditions.

In contrast to *Xanthomonas *species, *A. vinelandii *exhibits a very high level of respiration rate, up to 100 mmol O_2 _g _biomass_^-1 ^h^-1 ^[23,24]. This could explain why the main cellular responses, in terms of the growth rate and the alginate production, occur under very low DOT (from 1.0 to 5%) [9,10]. Therefore, it would be expected that a change in the agitation rate (and thus in the OTR and OUR) might cause a significant change in the bacterial response, even though the DOT in the bulk liquid is kept constant.

**Figure 6:**
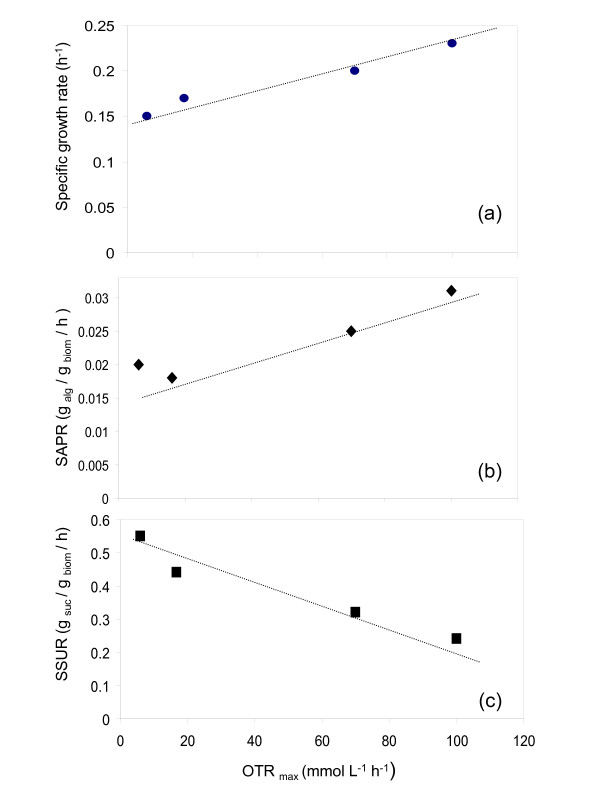
**Specific growth rate (a), specific alginate production rate (b) and specific sucrose uptake rate (c) in terms of the maximum oxygen transfer rate (OTR_max_) in cultures of *A. vinelandii *under oxygen-limited and non oxygen-limited conditions**.

Figure 6 shows a summary of the specific growth rate (μ), the specific alginate production rate (SAPR) and the specific sucrose uptake rate (SSUR) as a function of the OTR_max _obtained under the different conditions tested. An increase in the OTR_max _caused an increase in μ and in the SAPR, obtaining a maximal value of 0.23 h^-1 ^and 0.031 g _alg_^-1 ^g _biom_^-1 ^h^-1 ^when the OTR_max _was of 100 mmol L^-1 ^h^-1 ^for μ and SAPR, respectively. In contrast, for conditions leading to higher OTR_max _the sucrose uptake rate decreased significantly to values of 0.24 g _suc_^-1 ^g _biom_^-1 ^h^-1^.

The higher alginate production rate obtained when the cultures were conducted under conditions that resulted in higher OTR_max _might be explained considering that alginate synthesis needs a high energy input [25]. Therefore, in cultures where the respiration rate is high, the cells would have more available energy. It is known that the synthesis of polysaccharides represents a large portion of biosynthetic activity of the microbial cell. For example, it has been estimated that 3 moles of ATP were needed for the production of each mannuronic acid or guluronic acid residue in the polysaccharide [25].

Our results are in agreement with previous studies [9,10,13], which have found that the alginate synthesis rate and the specific growth rate increased at high agitation rate (600-700 rpm) with respect to the cultures of *A. vinelandii*, developed at 300 rpm and DOT of 5% of air saturation. Sabra et al. [10] found that in the cultures developed under low agitation rate, a slimy layer or alginate capsule is formed around the cells. They propose that this capsule could overcoming the oxygen stress and protect the nitrogenase system and in turn cause a decrease in the alginate biosynthesis. According to those authors, the thickness of this capsule was found to be strongly affected by the shear rate in the bioreactor. Under the light of the data reported in this paper, the differences, in terms of the alginate synthesis rate, could be explained considering the external mass transfer limitations determined by the oxygen transfer rate and the oxygen consumption rate, rather than due to diffusional limitations into the cells caused by a possible alginate capsule formed around cells.

The results shown in Figure 5 reveal important changes in the molecular mass of the alginate in terms of the OTR_max _of the culture. At low (6 mmol L^-1 ^h^-1^) and high OTR_max _(70-100 mmol L^-1 ^h^-1^) the alginate molecular mass was around 150-200 kDa.

It is possible that the cellular respiration influences the transcription of the genes *alg8, alg44, or algK*, which encode the polymerase complex. However, the mechanisms involved in polymerization and the depolymerization steps are still unknown. Previous studies by Díaz-Barrera et al [7,16] have shown the close relationship between the OTR_max _and the molecular mass of the alginate. In contrast to what we found in our study, Díaz-Barrera et al [7] reported that the mean molecular mass of the alginates increased in a monotonic way from 220 to 1560 kDa, when the OTR_max _was from 9 to 3.0 mmol L^-1 ^h^-1 ^(controlled by changes in the agitation). These differences could be due to the fact that in the study reported by Díaz- Barrera et al [7], the cultures were conducted without control of the DOT and under oxygen-limited conditions. Another possible explanation is that the range of OTR tested by those authors was lower than those tested in our study (~10-100 mmol L^-1 ^h^-1^). The same authors reported in chemostat cultures [16] developed under different OTR at a constant growth rate of 0.1 h^-1^, that the MMM of the alginate increased 850 to 1690 kDa, when the OTR was raised from 5.1 to 13 mmol L^-1 ^h ^-1 ^and dropping later to 1190 kDa when the OTR was increased at 14.5 mmol L^-1 ^h ^-1 ^.

It should be pointed out that in the two studies mentioned above [7,16] the MMM of the polymer was higher (up to 1690 kDa) with respect to the values obtained under oxygen controlled conditions (from 150 to 550 kDa). It is known that when *A. vinelandii *is cultured under non oxygen-controlled conditions (both in shake flasks and in stirred bioreactors) the mean molecular mass can reach values of up to 1900 kDa and viscosities up to 520 cps for broths containing about 5 g L^-1 ^of alginate [26]. However, in laboratory fermenters (1 L), in which pH and DOT were kept constant, the MMM and the viscosity of the broths obtained were considerably lower, obtaining alginates with a molecular mass of less than 650 kDa and viscosities lower than 100 cps for an alginate concentration approximately 5.0 g L^-1 ^[9,13]. The high molecular mass obtained in the alginates produced in shake flasks is related to the very low achievable OTR_max_, which is common to find in flasks operated at 200 rpm and 100 mL filling volume [6].

Although the role of the oxygen transfer rate in the polymerization of the alginate is not mechanistically understood, the results of the present study show for the first time the close relationship between the molecular mass of the alginate synthesized by *Azotobacter vinelandii *and the OTR_max _in cultures developed under non oxygen-limited conditions.

**Figure 7:**
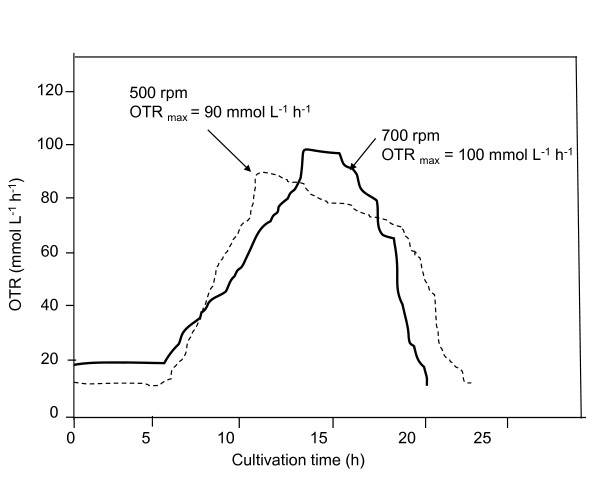
**Evolution of the oxygen transfer rate (OTR) during the cultivation of *A. vinelandii *in a 3.0 L bioreactor at 500 and 700 rpm and under constant DOT of 5%**.

In order to demonstrate that the OTR, and not the agitation rate (and therefore the high stress on the cells), was responsible for the changes in the alginate production and the molecular mass of the alginate and the alginate production, experiments at different agitation rate (500 and 700) and keeping constant DOT at 5% were conducted. As shown in Figure 7, an increase in the agitation rate (from 500 to 700 rpm) did not significantly affect the profiles of the OTR and OTR_max _values of the cultures. The OTR_max _was 90 mmol L^-1 ^h^-1 ^at 500 rpm, whereas in the cultures conducted at 700 rpm, the OTR_max _was 100 mmol L^-1 ^h^-1^. In spite of the changes in the agitation rate of the culture, the specific alginate production rate (SAPR) was similar for both conditions evaluated, achieving a maximum of 0.028 and 0.031 g _alg_/g _biom _h for 500 and 700 rpm, respectively. On the other hand, the mean molecular mass of the alginate isolated from the cultivations conducted at 500 and 700 rpm was very similar, reaching an average value of 160 ± 40 kDa. The previous results support the fact that the alginate yield and the molecular mass of the polymer seem to be associated to the OTR_max _value of the culture, independently of the agitation rate. It is known that alginate exhibits a high resistance to degradation by mechanical agitation and the mean molecular mass remains without changes, when the polymer is agitated up to 700 rpm during 24 h of incubation [9]. Thus, the agitation rate *per se *would not alter the molecular mass of the alginate. Another important point to consider is that *A. vinelandii *produces alginate lyases, which degrade the alginate. Therefore, it is possible that the low molecular mass observed in the cultures developed at 500 and 700 rpm is derived from the action of these enzymes, which degrade the polymer in the final stages of cultivation [13,15].

## Conclusions

Under oxygen-limited and non oxygen-limited conditions, the alginate production rate and the molecular mass of the polymer were closely associated to the OTR_max_, independently of the DOT of the culture. As the polysaccharides synthesis requires high energy levels, it would seem obvious the relationship between OTR_max _and the synthesis of alginate; however, the mechanisms involved in alginate polymerization remains to be elucidated. There is an optimal condition of OTR_max _under which alginate MMM was the highest and consequently this information can be used to establish an operational condition aimed to maximize the molecular mass of the alginate.

## Methods

### Microorganism

Experiments were carried out using the wild type *Azotobacter vinelandii *ATCC 9046. This strain was maintained by monthly subculture on Burk's agar slopes and was stored at 4°C.

### Inoculum preparation

*A. vinelandii *was grown in a modified Burk's medium, containing ammonium acetate (1.0 g L^-1^) [7]. Cultures were carried out in a 500 mL Erlenmeyer flask over a period of 24 h, containing 100 mL of culture medium at 200 rpm and 29°C in an orbital incubator shaker (shaking amplitude of 2.5 cm, New Brunswick Scientific Co., Model G 25).

### Bioreactor cultures

The cultures were carried out in a 3.0 L Applikon fermentor (Schiedam, The Netherlands) using a 2.0 L of working volume. Rusthon turbines with an impeller-vessel diameter ratio (D/T) 0.5 were used. Impeller spacing was of 0.05 m. Cultures were kept at a temperature of 29°C and the aeration was performed with a 7-orifice sparger at an airflow rate of 1.0 L min^-1^. The pH was measured with an Ingold (Columbus, USA) probe and controlled by an on/off system using a peristaltic pump and a 2N NaOH solution. Dissolved oxygen tension (DOT) was measured with an Ingold polarographic probe and controlled at 0.5 and 5% of the saturation. This was made by gas blending, automatically varying the proportions of nitrogen and oxygen in the inflowing gas through two 5850F mass flow controllers (Brook Instruments B.V. The Netherlands) and using a system based on a PID control which has been previously described [13].

The following proportional-integral-derivative control (PID) algorithm was used:

$${\text{O}}_{\text{2}}{\%}_{n}={\text{O}}_{\text{2}}{\%}_{n-1}+K_{\text{c}}[(\text{1}+(\text{T/}\tau_{\text{i}})+(\tau_{\text{d}}/T))\;E_{n}-(\text{1}+(\text{2}\tau_{d}/T))\;E_{n}\text{-1}+(\tau_{\text{d}}\;T)\;E_{n}\text{-2}]$$

where O_2_% is the percentage of aperture of the mass flow controller for oxygen, K_c_, τ_i_, and τ_d _are the proportional, integral and derivative constants, respectively, T is the discrete sampling time interval, the subindex *n *refers to the discrete sample, and E is the error of difference between the desired (setpoint) and measured DOT.

Therefore, the agitation rate could be varied without affecting the DOT concentration in the broth culture. Agitation rates of 300, 500 and 700 rpm were used. The bioreactor was inoculated with 200 mL of cells (2 g/L), grown previously in Erlenmeyer flasks. Cultivations were repeated three times and the figures show the mean values and standard deviations among replicas.

The oxygen transfer rate was determined by online measurements of O_2 _in the exit gas using a paramagnetic oxygen analyser (Teledyne Instruments, USA) [7,27]. The following equation was applied to calculate OTR from the exit gas analysis:

(1) $$OTR=\frac{\text{VG}}{{\text{V}}_{\text{L}}{\;\text{V}}_{\text{N}}}\left(Xin-Xout\right)$$

In which V_G _is the gas inlet flow rate (L h^-1^), V_L _the fermentation working volume (L), V_N _the molar volume (L mol^-1^) and Ҳ_in _and Ҳ_out _the mole fractions at gas inlet and outlet, respectively.

It is important to point out that OTR is a dynamic parameter that changes during cultivation. However, the maximum oxygen transfer rate, OTR_max _(used in the present study), is a fixed value that is only dependent on the operating conditions of the stirred bioreactor and, to a lesser extent, on the medium composition. The OTR value will always be smaller than or, at most, equal to the OTR_max _value.

### Analytical methods

#### Biomass, alginate, sucrose and ammonium concentration

Biomass and alginate concentrations were determined gravimetrically as described previously [9,13,26,28]. Sucrose was assayed for reducing power with a DNS reagent. Samples were previously hydrolysed by using β-fructofuranosidase (Gist Brocades) in order to generate glucose and fructose and were then assayed to determine the reducing power with DNS reagent [29]. The ammonium concentration was measured using the phenolhypochlorite method described by Kaplan [30]. The specific growth rate (μ) was calculated using the logistic model reported previously by Klimek and Ollis [31].

#### Mean molecular mass

The mean molecular mass of the alginate was estimated by gel permeation chromatography (GPC) with a serial set of Ultrahydrogel columns (UG 500 and Linear, Waters), using a HPLC system with a differential refractometer detector (Waters, 410). Further details of the technique are reported elsewhere [6,28].

## Competing interests

The authors declare that they have no competing interests.

## Authors' contributions

EL performed the experiments and was involved the analytical determinations. EG y CFP supervised EL experimental work and they were involved in the manuscript preparation and critical reading as well as in the design of the present study. All authors read and approved the manuscript.
